# Bacterial and Archaea Community Present in the Pine Barrens Forest of Long Island, NY: Unusually High Percentage of Ammonia Oxidizing Bacteria

**DOI:** 10.1371/journal.pone.0026263

**Published:** 2011-10-20

**Authors:** Vishal Shah, Shreya Shah, Murty S. Kambhampati, Jeffery Ambrose, Nyesha Smith, Scot E. Dowd, Kevin T. McDonnell, Bishnu Panigrahi, Timothy Green

**Affiliations:** 1 Department of Biology, Dowling College, Oakdale, New York, United States of America; 2 Department of Biology, Southern University at New Orleans, New Orleans, Louisiana, United States of America; 3 Research and Testing Laboratory, Lubbock, Texas, United States of America; 4 Department of Mathematics and Computer Science, Dowling College, Oakdale, New York, United States of America; 5 Brookhaven National Laboratory, Upton, New York, United States of America; University of Hyderabad, India

## Abstract

Of the few preserved areas in the northeast of United States, the soil in the Pine Barrens Forests presents a harsh environment for the microorganisms to grow and survive. In the current study we report the use of clustering methods to scientifically select the sampling locations that would represent the entire forest and also report the microbial diversity present in various horizons of the soil. Sixty six sampling locations were selected across the forest and soils were collected from three horizons (sampling depths). The three horizons were 0–10 cm (Horizon O); 11–25 cm (Horizon A) and 26–40 cm (Horizon B). Based on the total microbial substrate utilization pattern and K-means clustering analysis, the soil in the Pine Barrens Forest can be classified into four distinct clusters at each of the three horizons. One soil sample from each of the four clusters were selected and archaeal and bacterial populations within the soil studied using pyrosequencing method. The results show the microbial communities present in each of these clusters are different. Within the microbial communities present, microorganisms involved in nitrogen cycle occupy a major fraction of microbial community in the soil. High level of diversity was observed for nitrogen fixing bacteria. In contrast, *Nitrosovibrio* and *Nitrosocaldus spp* are the single bacterial and archaeal population respectively carrying out ammonia oxidation in the soil.

## Introduction

Microorganisms play an important role in the soil geology, hydrology, and ecology, and any change in microbial diversity can influence the soil quality and health [1]. Being at the bottom of the food chain, changes in microbial communities are often a precursor to the changes in the health and viability of the environment as a whole [2]. Our conceptual and predictive understanding of soil ecosystem processes, functions and management can be enhanced only upon obtaining the knowledge about the microbial community structure and composition in a given region. While many terrestrial and aquatic ecosystems have been studied for its microbial flora, no detailed report exists on understanding the microbial flora present in the soil of Pine Barren Forests in United States and evaluating their role in ecological cycles.

The vegetation known as the Pine Barrens, also known as an ecological desert, is scattered throughout the northeastern United States and beyond. Compared to vegetation in other forest types, the Pine Barrens is a unique region owing to the sandy, acidic, nutrient-poor soil made up largely of coarse sands and gravels deposited by recent withdrawal of glaciers [3]. The term “barrens” was coined by early settlers who unsuccessfully tried to raise their traditional vegetables and field crops in the sandy, acid soils of these regions [4]. Today, we know these areas are not really barren, for many forms of plant life- such as members of the pine family (Jack Pine, Red Pine, Pitch Pine), the beech family (Blackjack Oak and Scrub Oak) and the heath family (huckleberries, blueberries, cranberries) - do well in the highly acidic sandy soils [5]. However, these areas are still called barrens, a term that is used consistently in both popular and scientific references to these areas. A few characteristics of Pine Barrens soil are:

- The soil of the Pine Barrens is acidic. Pine and Oak trees drop litter composed primarily of needles and leaves. This litter is not readily digested by most microorganisms, decomposes slowly and accumulates on the soil surface. The decomposition by-products are strongly acidic and this makes the soil of Pine Barrens acidic, ranging from 4.0 to 4.5.
- Because of the acidic nature, the soil in the Pine Barrens contains high concentration of iron and aluminum. The cation exchange capacities are of extremely low order with a low base saturation [6].
- Fires are common in Pine Barrens and are necessary to maintain these regions as it replenishes the soil with nutrition; helps control insect infestation and dispersal of pine seeds [7].
- Water drains rapidly through layers of these porous soils to leave the surface droughty in spite of heavy rainfall in the region.

The Long Island Pine Barrens (LIPB) in New York is the second largest Pine Barrens in the country, next to the Pine Barrens in New Jersey. LIPB contains regionally rare wetland communities and rare upland communities including pitch pine-oak-heath woodland and the dwarf pine plains. The soil in the LIPB has all of the earlier mentioned characteristics. Besides, it is also exposed to the variation in temperature typical of Long Island. Long Island has warm, humid summers and cold winters. Average winter temperature is 0.2°C and the summer average is 22.2°C. Rainfall and snow averages are 42 inches and 30 inches, respectively. The microorganisms present in the LIPB have to be adapted to survive and flourish under such harsh conditions.

In the current study we illustrate the identification of the soils across LIPB that differ widely in their microbial community profile and also report the bacterial and archaea community structure present in the LIPB soil.

## Results and Discussion

**Figure 1:**
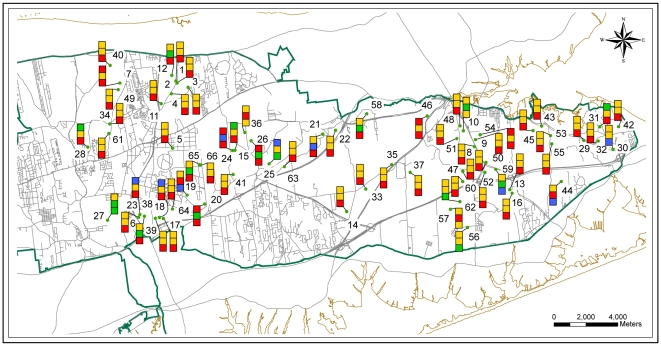
Clustering of the soil samples collected from 66 sampling locations of the Long Island Pine Barrens at three different horizons. Clusters are classified into four clusters according to their substrate utilization pattern. Color indicates cluster membership. The top square in each glyph indicates the cluster found at Horizon O, the middle square corresponds to Horizon A, and the bottom square to Horizon B.

**Figure 2:**
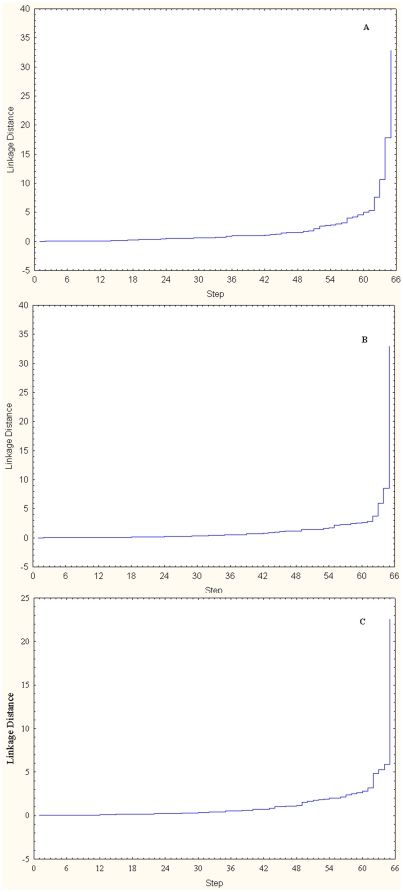
Amlagamation schedule used to identify the number of major clusters (K) for each horizon (based on total substrate utilization pattern). A, Horizon O, B, Horozon A, C, Horizon B.

Soil samples were collected from 66 sampling locations across the LIPB as illustrated in Figure 1 and Table 1 describes the types of vegetation present in each sampling plots. Hierarchical structure analysis (also called tree-like structure analysis) was performed using total community substrate utilization pattern obtained from the microbial community within each horizon. Ward's linkage method was employed as the algorithm of amalgamation because it uses an analysis of variance approach to calculate the distances between the clusters. The advantages of Ward's method include the approach being non-iterative and its ability to create clusters having of units having high degree of uniformity [8]–[9]. Figure 2 shows the amalgamation schedule graphs obtained for each of the three horizons. In the three graphs one can observe that after four big fusion steps, the linkage distance separating the two steps is very small (<1 unit). The decreasing linkage distance after fourth step suggests that the difference amongst the new clusters being formed is minimal and the soil from each of the three horizons can be distributed in four distinct clusters [10]. K-means clustering was then carried out to find the members of each of the four clusters for the three horizons and the results are described in Figure 1. By simple visualization of the figure it is clear that the members of each of the four clusters in the three horizons are scattered through out the LIPB. As can be seen in Table 1, the sixty-six sampling sites included 9 sites that have coastal oak vegetation, 24 oak-pine vegetation, 9 pine-oak vegetation, 3 scrub oak vegetation, 2 dwarf pine vegetation and 19 pitch pine vegetation. The difference between each of these vegetation types is the type of community, the relative abundance of pitch pines and scrub oaks in the area along with blueberry and huckleberry trees (Table 2). No correlation can be obtained between the clusters and the forest type. Similar results have been reported earlier by Fierer & Jackson [11]. On a cautionary note, it is possible that correlation could still exist between certain species of plants present and the microbial community present. However, as no detailed vegetation survey of LIPB has yet been published, such analysis was not carried out in the current study. No similarity was also found between the geographical location of the sites (north shore or south shore of the island) indicating that weather may not be the primarily factor in defining the microbial community in the soil of LIPB.

**Table 1:** Vegetation type in the sampling locations selected in the study.

Forest type	Sampling location no.
Pine Oak forest	1,2,24,29,30,36,38,39,63
Pitch Pine	3,4,8,9,10,32,34,37,46,47,48,51,52,53,54,55,64,65,66
Coastal Oak	5,6,7,11,12,14,19,20,27
Oak-Pine	13,15,16,17,18,21,22,23,25,26,28,31,33,35,40,41,42,43,44,45,49,50,58,59
Scrub Oak	56,57,61
Dwarf Pine	60,62

The Forest type data has been obtained from the Foundation for Ecological Research in the Northeast (FERN), who classified the forests based on field survey.

**Table 2:** Vegetation composition in various areas of Pine Barren Forests, NY.

Forest type	Community type	Presence of Pitch Pine	Presence of Scrub Oak	Presence of Blueberry and Huckleberry
Costal Oak	Forest	<10%	None	Continuous
Oak – Pine	Forest	11–49%	Scattered	Continuous
Pine – Oak	Forest	50–89%	Scattered	Continuous
Pitch Pine	Forest	90% or more	Continuous	Scattered
Pitch Pine Scrub	Shrub land	Primarily Pitch Pine with some Tree Oaks	Continuous	Scattered
Dwarf Pine	Shrub land	Pitch Pine and Dwarf Pine	Nearly continuous	Nearly continuous

The Data has been obtained from the Foundation for Ecological Research in the Northeast (FERN), who classified the forests type based on field survey.

**Figure 3:**
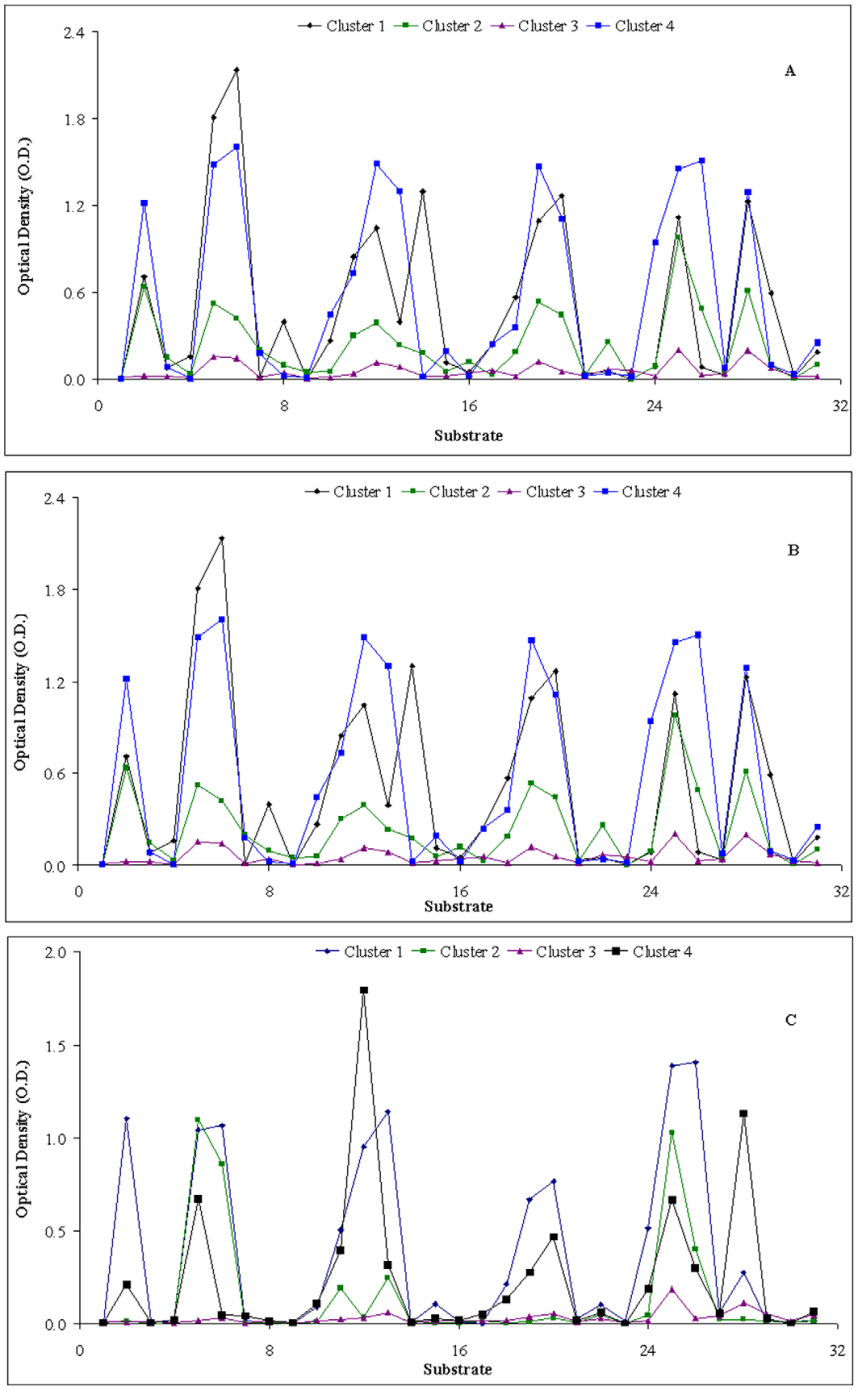
Graph of means of the optical density (O.D.) for different substrates. A, Horizon O, B, Horozon A, C, Horizon B. The substrates indicates on X axis: 1, β-Methyl-D-Glucoside; 2, D-Galactonic Acid Y-Lactone; 3, Xylose; 4, i-Erythritol L-Arginine; 5, D-Mannitol; 6, N-Acetyl-D-Glucosamine; 7, D-Cellobiose; 8, Glucose-1-Phosphate; 9, α-D-Lactose; 10, D,L-α-Glycerol Phosphate; 11, L-Arginine; 12, L-Aspargine; 13, L-Phenylalanine; 14, L-Serine; 15, L-Threonine; 16, Glycyl-L-glutamic Acid; 17, Phenylethylamine; 18, Putrescine; 19, Tween 40; 20, Tween 80; 21, α-Cyclodextrin; 22, Glycogen; 23, 2-Hydroxy Benzoic Acid; 24, 4-Hydroxy Benzoic Acid; 25, Pyruvic Acid Methyl Ester; 26, D-Galacturonic Acid; 27, γ-Hydroxybutyric Acid; 28, D-Glucosaminic Acid; 29, Itaconic Acid; 30, α-Ketobutyric Acid; 31, D-Malic Acid.

Analyzing the number of sites in a given cluster for all three horizons, it is clear that one cluster in each of the three horizons have majority of all the 66 sites (Table 3). Cluster 4 in horizons O and A have 52 and 51 sites respectively whereas cluster 1 in horizon B has 55 sites. A total of 37 sites are grouped within the same cluster all the three horizons. For the remaining 29 sites, there is no similarity on how they are clustered in the three horizons. For example, while site nos. 18, 23 and 25 form their own unique cluster in horizon O; they cluster with 48 other sites to form cluster 4 in horizon A and are not present within the same cluster for horizon B. Similarly, 21 and 24 are the only members of cluster 3 in horizon A where as they are clustered together with many other sites in horizon O and B. Euclidean distances between the centers of clusters for each horizon confirm the distinct nature of each cluster, with all clusters largely apart from each other (Table 3). Examination of the means for each cluster on each substrate further defines how distinct our 4 clusters are. Ideally, different means for most, if not all substrates should be obtained. Figure 3 displays the plots of means for each cluster within a given horizon and indeed the means vary for most of the substrates between the clusters. The raw data of the optical density with standard deviation are provided in Table S1. Interestingly, cluster 4 in horizons O and A and cluster 1 in horizon B has the lowest mean O.D. for almost all the substrates. It should be noted that all these clusters have more than 50 sites grouped together (Table 3). This would indicate that the microbial community present in these clusters does not have high affinity for the tested substrates. Other three clusters in all the horizons have varying degrees of affinity for different substrates and thus resulting in different cluster curves in the graph.

**Table 3:** Number of sites within each cluster in all the three horizons tested and the Euclidean distances between clusters.

Horizon O					
Cluster No.	No. of sites	Euclidean distances between clusters			
		1	2	3	4
1	6	0.000	-	-	-
2	5	0.524	0.000	-	-
3	3	0.470	0.473	0.000	-
4	52	0.751	0.353	0.698	0.000

Horizon A.					
1	4	0.000	-	-	-
2	9	0.387	0.000	-	-
3	2	0.383	0.628	0.000	-
4	51	0.644	0.276	0.868	0.000

Horizon B.					
1	55	0.000	-	-	-
2	4	0.571	0.000	-	-
3	4	0.417	0.447	0.000	-
4	3	0.299	0.419	0.429	0.000

One sample from each of the four clusters across the three horizons was selected for chemical analysis and to elucidate the microbial diversity present in the soil of Pine Barrens Forest using pyrosequencing analysis. Chemical analysis of soil samples were performed (Table 4). Results show that the concentration of iron and aluminum increase as we go deep into the soil while total organic carbon (TOC) and total Kjeldahl nitrogen (TKN) decreases as we go deep. Indeed, comparing the values of the representative samples from each cluster for a given horizon, the differences are evident. We propose that the similarities in the cluster members could be reflected in their soil properties.

**Table 4:** Chemical properties of the soil representative of cluster within the horizon.

Sample ID	pH	TOC	TKN	Al	Fe
		(g/Kg)	(g/Kg)	(g/Kg)	(g/Kg)
26 O	4.94	20.9	1.1	0.8	1.3
15 O	4.41	14.9	1.0	0.7	0.9
23 O	4.31	37.1	1.5	1.2	1.5
13 O	4.48	25.2	4.4	0.3	0.4
26 A	4.78	BDL	0.4	1.3	2.2
44 A	4.68	4.1	0.4	0.9	1.3
21 A	4.64	6.6	0.3	1.9	2.8
13 A	4.8	1.4	0.1	0.3	0.5
44 B	4.95	4.3	0.2	2.1	3.0
25 B	4.69	5.4	0.2	2.8	4.2
10 B	4.98	4.9	0.2	2.2	2.5
13 B	4.74	4.0	0.1	2.3	4.3

**Figure 4:**
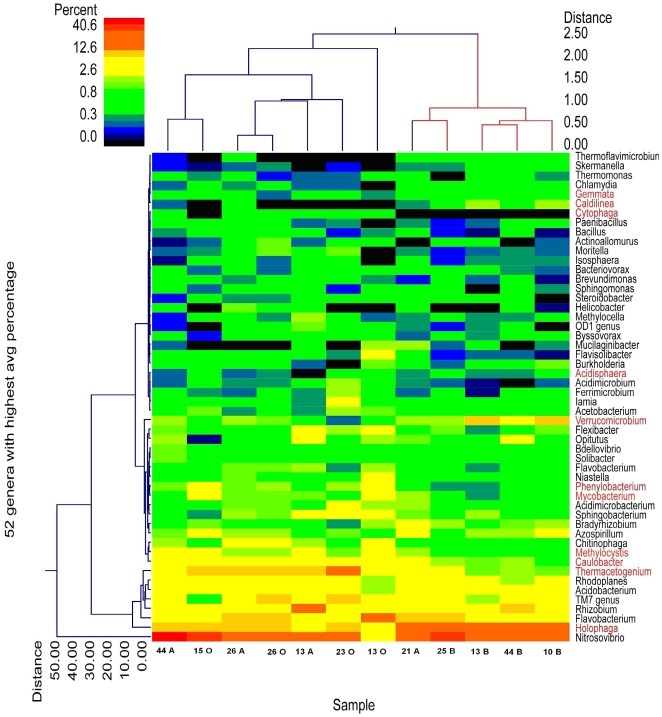
Dual hierarchal dendrogram based upon top 52 genera classified using bacterial tag-encoded FLX-titanium amplicon pyrosequencing. Clustering for genera and for samples are based upon Ward's minimum variance and with Manhattan distances. Genera are colored red based upon differences derived from ANOVA with Tukey-Kramer post hoc analysis to give a general overview of notable differences between horizons. The heatmap represents the relative percentage of each genera within each sample with legend presented at the top left of the figure.

Taxonomically, all the soil samples had bacterial population from 11–17 phylum, with the samples from top horizon containing the least number of phylum (Table S2). Bacteria belonging to Actinobacteria, Proteobacteria, Bacteroidetes and Acidobacteria were the most prominent phylum present. Reads belonging to TM7, Verrucomicrobia, Firmicutes, Planctomycetes, Chlamydiae, Deinococcus-Thermus, Cyanobacteria, chloroflexi, candidates phylums OD1 and OP10, Gemmatimonadetes, Nitrospirae and Elusimicrobia were found to be minor groups. Table 5 shows the microbial diversity within the soil at genus level for all the twelve samples tested (Table S3 provides the complete diversity including that of the minor populations). Figure 4 also provides a broad overview of the data based upon the top 52 genera. BLAST results clearly suggest that the organisms involved in nitrogen metabolism occupy a major fraction of the microbial community in the soil of Pine Barrens Forest. Members of *Nitrosovibrio*, *Flavobacteria*, *Rhizobium*, *Bradyrhizobium*, *Verrucomicrobium* and *Azospirillium* genus are the major organisms that are present in the studied soil and are known in literature to be involved in nitrogen cycling. Thus, with members of *Flavobacteria*, *Rhizobium*, *Bradyrhizobium*, *Verrucomicrobium*, *Azospirillium*, classified within this analysis there is likely enough diversity to support the nitrogen fixation. Surprising, only *Nitrosovibrio* genus is the ammonia oxidizing bacteria present that is able to convert ammonia to nitrates and nitrites. In addition while bacterial ammonia oxidizers (AO) usually comprise <1% of the total bacterial community present in normal soil [12]–[13], in Pine Barrens Forest they were as high as 41%. Genera in the archaeal genera *Nitrosocaldus* are also known AO organisms and as seen in Table 5, they are also present in relatively high percentage in lower horizon, while *Hyperthermus* and *Thermoplasma* were the other Archaea classified.

**Table 5:** Percentage of rDNA sequences of bacteria and archae present in the soil at various horizons and locations in the Pine Barren Forest.

Sampling location	13O	15O	23O	26O	13A	21A	26A	44A	10B	13B	25B	44B
**Bacteria**												
*Acetobacteraceae (genus)*	1	1	2	0	0	1	0	1	0	0	0	0
*Acidimicrobiaceae (genus)*	2	1	5	1	1	1	1	0	1	0	1	1
*Acidimicrobium*	0	0	1	0	0	0	0	0	0	0	0	0
*Acidobacteriaceae (genus)*	2	2	4	2	1	3	1	2	8	3	4	4
*Acidobacterium*	1	2	4	3	3	4	3	3	3	4	5	4
*Azospirillum*	0	2	1	1	1	2	2	1	2	2	1	2
*Bradyrhizobium*	1	1	0	0	1	2	1	1	2	1	1	1
*Burkholderia*	1	0	0	1	0	0	0	0	1	0	0	0
*Caldilinea*	0	0	0	0	0	0	0	0	2	1	1	0
*Caulobacter*	5	3	2	3	2	2	4	2	1	1	2	1
*Chitinophaga*	3	1	1	3	2	0	2	1	0	1	1	0
*Chlamydiales (genus)*	0	0	0	0	1	1	1	1	1	0	1	2
*Ferrimicrobium*	1	0	2	0	0	0	0	0	0	0	0	0
*Flavisolibacter*	3	0	0	0	0	0	0	0	0	0	0	0
*Flavobacteria (genus)*	20	3	2	7	2	7	7	3	6	5	6	2
*Flavobacterium*	2	0	0	1	2	0	1	1	0	0	0	0
*Flexibacter*	2	0	1	0	3	1	1	1	1	0	1	1
*Helicobacter*	0	0	0	0	1	1	1	0	0	0	0	0
*Holophaga*	3	6	7	6	5	10	8	7	15	17	12	14
*Iamia*	0	0	2	0	0	0	0	0	0	0	0	0
*Methylocystis*	2	3	1	2	2	2	1	2	0	1	1	1
*Moritella*	0	0	1	1	0	0	1	0	0	0	0	0
*Mucilaginibacter*	1	0	0	0	0	1	0	0	0	0	0	0
*Mycobacterium*	2	3	1	1	1	1	1	1	1	0	0	1
*Niastella*	3	1	0	1	0	1	1	1	1	0	0	0
*Nitrosovibrio*	4	38	19	21	23	24	21	41	17	27	35	26
OD1 (genus)	0	0	0	1	1	0	1	0	0	0	0	0
*Opitutus*	2	0	0	1	6	1	1	1	0	0	0	2
*Phenylobacterium*	2	2	1	1	0	1	1	1	0	0	0	0
*Planctomycetacia (genus)*	0	0	0	0	1	1	0	0	1	1	0	1
*Planctomycetales (genus)*	1	0	0	1	0	1	0	1	0	0	0	0
*Rhizobiales (genus)*	1	1	0	0	1	0	0	1	0	0	0	1
*Rhizobium*	5	4	3	4	9	5	3	4	6	4	3	6
*Rhodoplanes*	1	5	3	3	2	3	4	2	2	3	3	2
*Spartobacteria (genus)*	0	0	0	0	0	1	0	0	3	2	1	2
*Sphingobacterium*	3	0	2	1	2	1	1	1	1	1	0	0
*Thermacetogenium*	4	7	13	6	7	3	6	2	1	1	2	1
TM7 (genus)	3	1	6	8	3	2	6	3	3	1	2	5
*Verrucomicrobiales (genus)*	1	1	1	1	1	1	1	1	2	1	1	1
*Verrucomicrobium*	0	0	0	1	1	2	1	1	7	6	2	4
**Archae**												
*Nitrosocaldus*	0	0	11	0	0	9	50	0	100	35	86	86
*Hyperthermus*	100	100	89	100	100	91	50	100	0	65	14	0
*Thermoplasma*	0	0	0	0	0	0	0	0	0	0	0	14

The soil sampling locations are shown in Figure 1. O, 0–10 cm; A, 11–25 cm; B, 26–40 cm.

Occurrence of such high levels of AO, let alone from a single genus of bacteria and a single phylum of archaea have not been reported in the literature for any samples obtained from terrestrial ecosystem. While the reason for such high percentage of AO in the soil is not clear, we hypothesize that the major function of this organisms in Pine Barrens soil is to maintain the pH of the soil. Table 3 shows that the organic carbon levels in the soil are high, primarily because of the litter from the vegetation. The degradation of the organic matter and the activity of nitrogen fixing organisms would increase the level of ammonia in the soil. Indeed, the total nitrogen levels (organic and ammonia N) in the soil are high (>100 mg/Kg, Table 3) in all the samples with the levels highest in the top soil. If ammonia is allowed to accumulate in the soil the pH of the soil would rise. The microbial and vegetation communities in the Pine Barrens Forest have adapted to the acidic conditions and any increase in pH would likely alter the balance of the ecosystem. As the soil is sandy in nature, it has very poor buffering capacity making it more essential to continually remove ammonia from the soil. Presence of high concentration of *Nitrosovibrio* ensures immediate removal of ammonia, maintaining the acidic pH. The conversion of ammonia to nitrates would leave very little nitrogen in the soil as they would continually leach out of the soil. Only the vegetation that is able to survive in low nitrogen conditions with mechanisms allowing them to acquire adequate nitrogen from the soil, at a rate faster than the ammonia oxidizing bacteria, would thrive and flourish in the Pine Barrens. As normal vegetation requires high levels of nitrogen to grow, such populations are not found to thrive in the Pine Barrens. The Oak and Pine trees along with blueberries and huckleberries shrubs are predominant and are known to thrive under low nitrogen conditions. The recent conclusion of Yao et al. [14] stating that certain members of Pine trees have preference for nitrate and are not well adapted to ammonium-N as a sole nitrogen source regardless of the growth medium pH further supports our hypothesis.

To further generalize the microbial diversity in the soil of Pine Barrens, the bacterial data in Table 5 were collectively analyzed. As can be seen in Table 6, *Holophaga*, *Verrucomicrobium*, *Methylocystis*, *Mycobacterium Phylobacterium*, *Spartobacterium*, *Planctomyces*, *Caldilinea*, *Gemmata* and *Chloroflexus* all increase in average percentage with depth, while *Thermatogenium* and *Caulobacter* decreased with depth. Diversity estimates based upon rarefaction, Ace and chao1indicated there were no significant difference (using two-tailed T-tests) between the horizons. The O horizon samples averaged 1243 OTU based upon rarefaction at the 3% divergence, A at 1251 and B averaging 1153.

**Table 6:** Genera with significant differences across horizons.

Category	O	A	B
*Holophaga*	5.5^B^	7.6^B^	14.6^A^
*Thermacetogenium*	7.5^B^	4.6^AB^	1.4^A^
*Caulobacter*	3.3^B^	2.4^AB^	1.2^A^
*Verrucomicrobium*	0.5^B^	1.3^B^	4.6^A^
*Methylocystis*	0.6^B^	1.8^AB^	2.1^A^
*Mycobacterium*	0.4^B^	1^AB^	1.5^A^
*Phenylobacterium*	0.2^B^	0.7^B^	1.6^A^
*Spartobacterium*	0.03^B^	0.4^AB^	1.7^A^
*Planctomycetes*	0.03^B^	0.5^AB^	0.6^A^
*Caldilinea*	0.008^B^	0.1^B^	1.0^A^
*Gemmata*	0.2^B^	0.3^AB^	0.6^A^
*Helicobacter*	0.4^A^	0.1^B^	0.1^AB^
*Acidisphaera*	0.4^A^	0.1^B^	0.2^AB^
*Bacteriovorax*	0.1^A^	0.4^B^	0.2^A^
*Geobacter*	0.1^B^	0.3^A^	0.1^AB^
*Chloroflexus*	0.02^B^	0.03^B^	0.3^A^
*Cytophaga*	0.3^B^	0.3^B^	0^A^

Significant differences based upon uncontrolled ANOVA-tukey-kramer analysis are indicated with standard notation. For instance Holphage in the B horizon is significantly higher than in the A or the B horizon. Thus, across Genera cells that share a common letter are not significantly different cells that do not share a common letter are significantly different (P<0.05).

As the samples used for diversity studies covers all the distinct clusters, there is a high level of confidence in the generalization of the current results of microbial diversity to the entire Pine Barrens Forest in Long Island, New York. Based on the microbial diversity data, the future direction of research should involve evaluating the possibility of inhibiting the AO organisms in the soil and investigating the ability of agriculturally important vegetation to grow under such conditions. It could be possible that such steps could allow one to reclaim vast amount of currently barren lands having conditions similar to that of Pine Barrens Forest. We also urge fellow colleagues to join us in investigating the ecological significance of the organisms involved in nitrogen cycle at regional and global level, along with increasing efforts to isolate and characterize such organisms.

## Materials and Methods

### Sample collection

Soil samples were collected from 66 sampling locations across the LIPB as illustrated in Figure 1. No permits were required to obtain the samples. Table 1 describes the types of vegetation present in each sampling plots. The locations were randomly selected using a Geographic Information system (GIS) ensuring that the locations are spread across the LIPB and covering all the vegetation types. The locations of sample collections were confirmed by the use of Thales/Magellan Global Positioning System unit (GPS) MobileMapper CE. All sampling locations were more than 50 meters from disturbed areas such as roads, wetlands and other plots. The protocols of the safety of data collection were rigorously followed as recommended by the report of the U.S. Fish and Wildlife Services and the Foundation for Ecological Research in the North East [15]. At each location, soil samples were collected from three horizons: 0–10 cm (Horizon O); 11–25 cm (Horizon A) and 26–40 cm (Horizon B). Throughout the study, temperature of the soil was measured on-site. The soil temperatures ranged from 16°C to 26°C. The chemical analysis of the soil samples were performed by Long Island Analytical Laboratories, Holbrook, NY.

### BIOLOG ® Ecoplates

For analyzing the total community substrate utilization pattern of the soil, the method described by Kumar *et al.* was followed [16]. In brief, 1 g soil samples were dispersed in 9 mL sterile distilled water and after vortexing the mixture for 5 minutes, the solution was allowed to sit for 1 minute. 100 µl of the solution was then added to 9.9 mL of sterile distilled water and the solution was mixed for 1 minute. 100 µl of the diluted solution was added to each well of the 96-well Biolog® Ecoplates. The plates were incubated at 30°C for 48 h and the color formation (Optical Density, O.D.) in the Ecoplates was read using Tecan Microplate reader at 590 nm.

#### DNA extraction and TEFAP analysis

DNA was extracted from the soil using PowerSoil™ DNA Isolation Kit (MO BIO Laboratories, Inc., Carlsbad, CA) as per the supplier's instructions. Data on the microbial communities present in the soil was obtained by carrying out pyrosequencing analysis on the DNA. The microbial tag-encoded FLX amplicon pyrosequencing (TEFAP) was performed using primers Gray28F 5′ GAGTTTGATCNTGGCTCAG and Gray519r 5′ GTNTTACNGCGGCKGCTG for bacterial populations. For amplification of Archaeal populations the primers A340F90 5′ GYGCASCAGKCGMGAAW and a806R96 5′ GGACTACVSGGGTATCTAAT were used. Sequence was performed at Research and Testing Laboratory (Lubbock, TX) as has been described previously [17]–[18]. Following sequencing, all failed sequence reads, low quality sequence ends and tags and primers were removed along with the sequences collections depleted of any non-bacterial/fungal ribosome sequences and chimeras [19]–[20]. To determine the identity of microorganisms in the remaining sequences were assembled into clusters with uclust (www.drive5.com) and queried using a distributed BLAST (www.krakenblast.com) algorithm [21] against a comprehensive database of high quality rDNA sequences derived from NCBI (01-01-11) and evaluated as described previously [19]–[20], [22]–[23]. Unifrac analysis [24] to generate weighted distance matrices were evaluated using principal component analysis and rarefaction analysis was performed using Mothur [25] as described previously [19]–[20], [22]–[23]. Two tailed T-test was utilized to evaluate the significance of rarefaction data. Genera were evaluated using ANOVA with Tukey-Kramer Post hoc analysis. Dual hierarchal dendrograms based upon Ward's minimum variance and Manhattan distances were generated using NCSS 2007.

### Statistical methods

The Biolog® EcoPlate contains 31 carbon sources for soil community analysis and each of these 31 carbon sources are repeated 3 times in the 96-well plate. Mean of the triplicate absorbance values was calculated and used for further analysis. Net absorbance value for each substrate was calculated by subtracting the absorbance value of control (no substrate) from the absorbance value of well containing respective substrate. When the net absorbance values were negative, it was calculated as zero.

Garland recommended that the data obtained using Biolog® Ecoplates, be normalized prior to analysis [26]. However, in the current study (as discussed later), there are numerous samples where no color formation or little color formation was observed in any of the substrates present in the Ecoplates. Normalizing the data using the protocol described in Garland magnified the differences between the absorbance but did not significantly change the clustering results. Thus in the current study, no normalization step was included in the statistical analysis.

The goal of the clustering analysis was to reorganize the sampling locations into relatively homogenous groups based on their total community substrate utilization pattern. Cluster analyses of the data were carried out using Statistica (Release 8.0) software. The analyses were performed in sequential order as described below:

1. Tree cluster analysis was first carried out selecting Ward's method as the amalgamation rule and the distance measured as Euclidean units. In Ward's method the cluster membership is assessed by calculating the total sum of squared deviations from the mean of a cluster. Results of the analysis yielded hierarchical tree plots and amalgamation schedule. In a hierarchical analysis, increasingly dissimilar clusters must be merged as the cluster fusion process continues. Consequently, the classification is likely to become increasingly artificial as one goes along the fusion process. When one looks at the amalgamation schedule graph, it is possible to obtain the number of major steps of fusion after which the graph is significantly ‘flattened’ (the depth of each step is very small), suggesting that not much new information is portrayed by the following mergers of clusters. The number of distinct steps tells us out how many homogenous groups (K) are present in the result of clustering study. One can choose fine steps or steep steps depending on the resolution between the samples types desired. This amalgamation schedule obtained here is analogous to the ‘scree test’ of factor analysis [10].
2. Using K-means clustering, the 66 sampling sites were divided into K clusters by selecting the number of iterations as 10 and the initial cluster centers to be chosen to maximize initial between-cluster distances. The output of this step is the list of sites that are present in each cluster.

Ferrier et al. [27] proposed such two step clustering approach when classifying a space when employing using multiple data set. The advantage of the two-step clustering approach described here is that it relies on classification based on biological similarities and dissimilarities. Using other available approaches of modeling, we believe it would be possible to integrate other biological information such as vegetation pattern and multiple sets of environmental data into the data analysis to obtain an integrative classification of the space being studied.

Differences in the chemical properties of the soil were compared by obtaining the *p* - values using Spearman rank order correlation test. A value of ≤0.05 indicates a significant difference between the values.

## Supporting Information

Table S1 **Mean optical density for each substrate along with standard deviation.** (A, Cluster O; B, Cluster A; C, Cluster B.).
(XLS)

Table S2 **Bacterial population present in the soil of Pine Barrens Forest at each sampling horizon classified at phylum level.**
(XLS)

Table S3 **Bacterial population present in the soil of Pine Barrens Forest at each sampling horizon classified at genus level.**
(XLS)
